# Recovery of Peripheral Nerve with Massive Loss Defect by Tissue Engineered Guiding Regenerative Gel

**DOI:** 10.1155/2014/327578

**Published:** 2014-07-03

**Authors:** Shimon Rochkind, Zvi Nevo

**Affiliations:** ^1^Division of Peripheral Nerve Reconstruction, Department of Neurosurgery, Tel Aviv Sourasky Medical Center, Tel Aviv University, 6 Weizmann Street, Tel Aviv 64239, Israel; ^2^Department of Human Molecular Genetics and Biochemistry, Sackler School of Medicine, Tel Aviv University, Tel Aviv 6997801, Israel

## Abstract

*Objective*. Guiding Regeneration Gel (GRG) was developed in response to the clinical need of improving treatment for peripheral nerve injuries and helping patients regenerate massive regional losses in peripheral nerves. The efficacy of GRG based on tissue engineering technology for the treatment of complete peripheral nerve injury with significant loss defect was investigated. *Background*. Many severe peripheral nerve injuries can only be treated through surgical reconstructive procedures. Such procedures are challenging, since functional recovery is slow and can be unsatisfactory. One of the most promising solutions already in clinical practice is synthetic nerve conduits connecting the ends of damaged nerve supporting nerve regeneration. However, this solution still does not enable recovery of massive nerve loss defect. *The proposed technology* is a biocompatible and biodegradable gel enhancing axonal growth and nerve regeneration. It is composed of a complex of substances comprising transparent, highly viscous gel resembling the extracellular matrix that is almost impermeable to liquids and gasses, flexible, elastic, malleable, and adaptable to various shapes and formats. *Preclinical study* on rat model of peripheral nerve injury showed that GRG enhanced nerve regeneration when placed in nerve conduits, enabling recovery of massive nerve loss, previously unbridgeable, and enabled nerve regeneration at least as good as with autologous nerve graft “gold standard” treatment.

## 1. Introduction

Peripheral nerve injuries represent a major cause for morbidity and disability and pose substantial costs for society from a global perspective. The patients with peripheral nerve injuries acquire lifelong disability and require posttrauma peripheral nerve rehabilitation treatments following a growing number of traffic and work accidents, natural disasters, and military activity that cause disability associated with loss of sensory and motor functions and, in some cases, intractable pain. Recovery following severe peripheral nerve injury is often dismal despite the inherent capability for axonal regeneration.

Many severe peripheral nerve injuries can only be treated through surgical reconstructive procedures. The gold standard autograft repair of the damaged peripheral nerve is far from being optimal and often disappointing [1]. A major disadvantage of nerve autograft is the need to retrieve donor material from the patient, with added morbidity and concomitant loss of function. Significant prolonged disability and socioeconomic dependency are inevitable.

It is widely documented that poor outcome is reflected by microsurgical failure to adequately address nerve regeneration at the cellular level. This is compounded by insufficient autograft being available for major reconstruction. One of the promising solutions already in clinical practice is synthetic nerve conduits connecting the ends of damaged nerve, enabling nerve regeneration [2–5]. There are many advantages for using artificial nerve conduits in comparison to autologous nerve graft, explaining the efforts invested in optimizing this solution worldwide. Among the advantages are the following: procedure is simpler, there is a significant decrease in time of surgery, and finally, no sensation loss or cosmetic defect in leg exists. The disadvantage of artificial nerve conduits is the inability to bridge more than 2 cm long nerve loss. Therefore, the repair and regeneration of peripheral nerve injuries with massive loss defect is a major clinical issue for the relatively new fields of regenerative medicine, biomaterials, and tissue engineering.

The scientific and clinical communities are waiting for innovative therapies to be successfully applied in this field of medicine. Successful outcome will have a substantial impact on patient care, lifelong health, and wellbeing. The goals described herein are aimed to develop new devices, either alone or in combination with growth gels and cell therapy following massive loss to peripheral nerve.

The idea to develop gel for nerve regeneration originated from preliminary results that we received using previous generation of the gel [2, 3]. In an animal study (in rats) a polymer tube filled with gel was used for reconnection of completely transected peripheral nerve [2]. We found evidence of axonal sprouting through the gap inside the tube in the peripheral nerve with massive loss defect.

The proposed technology for supporting nerve growth in artificial nerve conduits (tubes) for treatment of peripheral nerve injury with massive loss defect is a biocompatible and biodegradable Guided Regeneration Gel (GRG) that enhances axonal growth and nerve regeneration. It is composed of a complex of substances comprising transparent, highly viscous gel that is almost impermeable to liquids and gasses, flexible, elastic, malleable, and adaptable to various shapes and formats. The gel resembles the extracellular matrix (ECM) and was found to support three dimensional growth and differentiation of various cell types including neuronal precursor cells, neurons, and neuronal accompanying cells. The proposed combination of GRG filling the nerve conduits is expected to provide an alternative to an autologous nerve graft, by supporting and enhancing axonal regeneration across a nerve gap, enabling reconnecting massive nerve gaps with nerve conduits.

## 2. Material and Methods

Double-blind randomized study on 32 Wistar rats was performed in order to evaluate the efficacy of proposed GRG gel, by using nerve guide conduits in complete peripheral nerve injury with 15 mm segmental loss.

### 2.1. GRG Preparation

Guided Regeneration Gel (GRG) is made of three components:

- *Hyaluronic acid* (HA, hyaluronan) that is highly hydrated and contributes to the success of the implant to survive the initial period of integration and its growth and regeneration by providing the proper hydration protecting from drying, as well as serving as an additional antioxidant agent to the SOD, protecting against oxygen stress. HA also serves as a reservoir—a vehicle to carry various agents and enabling their slow release. The typical richness in the content of hyaluronic acid in fetal tissues, together with the significant presence of stem cells, dictates the characteristic rapid and smooth healing of fetal wounds without scars.
- *The 16 amino acid peptide simulating laminin activities* contains two sequences of two pentapeptides found in laminin and shown as biologically active on various cell types. For neurons theses peptides are guiding their migration, differentiation, regeneration, and survival.
- *The enzyme-protein-long peptide, sodium dismutase* is a strong recombinant antioxidant protecting against oxygenative stresses that works better in combination with HA.

#### 2.1.1. Preoperative Preparation

Animals were housed with two animals per cage, in standard cages, and fed standard chow and watered. All rats were induced under general anesthesia with an intraperitoneal injection of xylazine (15 mg) and ketamine (50 mg). Depilation of the surgical site was accomplished with an electric animal clipper. Procedures were performed in a sterile manner in a room reserved for aseptic survival surgery.

### 2.2. Blinding

The animals were not marked prior to surgery. Each animal was given a unique ear mark before recovery from anesthesia.

### 2.3. Experiment Design

The left sciatic nerve was exposed and separated from the biceps femoris and semimembranosus muscles. The sciatic nerve was transected and a 10 mm nerve segment was removed.

**Figure 1:**
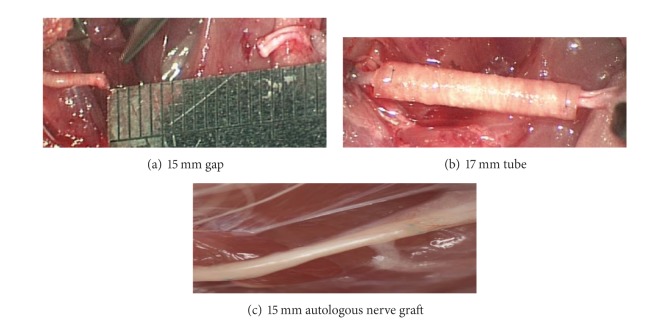
(a) Creating 15 mm segmental loss nerve damage; (b) nerve reconstruction using 17 mm tube; (c) nerve reconstruction using 15 mm autologous nerve graft.

A 17 mm conduit (tube) the NeuraGen hollow tube was placed between the proximal and the distal parts of the transected nerve for reconstruction enabling the nerve to enter the tube 1 mm each side providing a 15 mm gap between the proximal and distal end (Figure 1(a)). A 10 mm instead of 15 mm of nerve was removed, leaving longer proximal and distal parts. That was done in order to preserve elasticity of the nerve, which has allowed avoidance of tension during leg movement. The conduit (tube) was filled with GRG to align both nerve ends (Figure 1(b)). Controls included autologous nerve graft transplant (gold standard) (Figure 1(c)); empty tubes; and tubes filled with hyaluronic acid (HA). Two 9-0 nonabsorbable sutures were used for encoring of the tube to the epineurium at the proximal and distal nerve stamp. The muscular, subcutaneous, and skin layers were closed as standard.

The peripheral nerve injury was treated according to one of the treatments listed in Table 1 and followed for 90 days.

The study design was as shown in Table 1.

### 2.4. Histological Evaluation of the Operated Nerve

The rats were sacrificed by injecting lethal doses of xylazine and ketamine 90 days following the surgical procedure. The operated sciatic nerve was exposed.

Peripheral nerves fixed in 2.5% glutaraldehyde in Cacodylate bugger, ph 7.4 for overnight and postfixed in 1% osmium tetroxide for one hour. Following dehydration in a graded ethanol series and propylene-oxide, they were embedded in Eponate 12 Resin (glycerol polyglycidyl ether). Semithin sections were stained with Methylene Blue.

Histological slides were performed at three sites: 5 mm proximal to the tube/autograft, at the tube/autograft, and 5 mm distal to the tube/autograft. Axons were evaluated according to the followed histological score.

#### 2.4.1. Histological Score (Blind Examination)

(5) Similar to proximal part of the nerve;
(4) good amount of large-diameter axons;
(3) good amount of axons;
(2) moderate amount of axons;
(1) mild amount of axons;
(0) scar tissue (no axons).

The study received the local Helsinki approval (IRB) for animal research. It was performed in a double-blind and randomized design. The rat group affiliation was disclosed only after histological analysis for each nerve had been completed.

### 2.5. Analysis and Statistics

Statistical analysis and calculations were done using nonparametric statistics (Mann-Whitney *U* test and Kruskal-Wallis test).

Histological scores were measured in four groups.

## 3. Result

**Figure 2:**
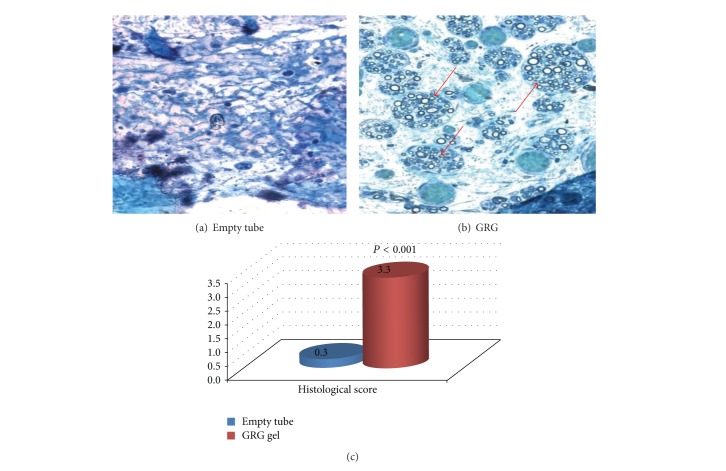
(a) No axons, connective scar tissue; (b) massive growth of regenerative axons into the tube. The graph reflects histological score of the distal part of the nerve (blind examination) in difference between amounts of axons in the GRG group (good amount of axons with tendency to large-diameter axons) versus empty tube (scar tissue—no axons).

**Figure 3:**
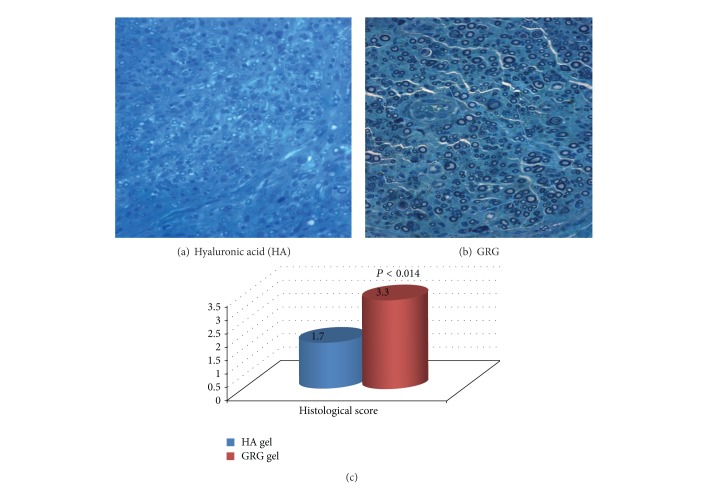
(a) Regenerated small axons in the HA treated group. (b) Regenerated axons in the GRG treated group (increased quality and quantity). The graph reflects histological score of the distal part of the nerve (blind examination) in difference between amounts of axons in the GRG group (good amount of axons with tendency to large-diameter axons) versus HA group (mild to moderate amount of axons).

Histological observation of the nerve showed no axonal growth into the tube in the empty tube reconstruction group (Figure 2(a)). In the group treated with tube filled with GRG, growth of myelinated axons was seen in the place where nerve defect was replaced by composite nerve transplant (Figure 2(b)), and continuation of axonal sprouting through the place of the tube to the distal part of the nerve (*P* < 0.001) was observed. The growth of myelinated axons through the tube to the distal part of the nerve was significantly enhanced (*P* < 0.014) as compared with group where tube was filled with HA (Figure 3).

**Figure 4:**
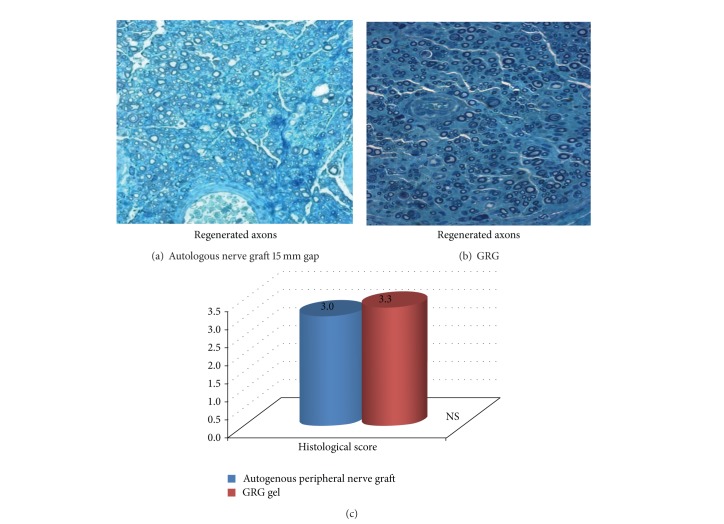
GRG gel is shown to enable nerve regeneration similar to autologous nerve graft reconstruction (gold standard). The graph reflects histological score of the distal part of the nerve (blind examination) in difference between amounts of axons in the GRG group (good amount of axons with tendency to large-diameter axons) versus autologous nerve graft group (good amount of axons).

The histological pictures of the GRG group versus autologous nerve transplanted group shows that no significant differences were found between both groups (Figure 4). GRG enabled optimal axonal regeneration as compared to gold standard.

## 4. Discussion

Peripheral nerve injury is of high consequence to civilians suffering from motor vehicle, work injuries, and acts of violence as well as to military personnel injured while serving their country. These injuries lead to partial or complete paralysis, severe pain, disabilities, and deterioration in quality of life. Many severe peripheral nerve injuries can only be treated through surgical reconstructive procedures. Complete peripheral nerve injury (PNI) always requires surgical procedure to reconnect the nerve. For major nerve loss, the current available option is autologous nerve grafts that are considered the gold standard for treatment in these cases. Such procedures are most challenging, since functional recovery is slow, and often unsatisfactory results occur with the inherent morbidity of the donor site.

The alternative solution, already in clinical practice, is synthetic nerve conduits connecting the ends of damaged nerve. Most repair scaffolds consist of a hollow tube made of polymeric materials such as silicone, biologic materials such as collagen, chitosan, or biodegradable polymers [1–11]. However, this solution enables optimal healing only when gaps are minimal. Existing nerve guides are poor at supporting regrowth and not designed to actively stimulate Schwann or neuronal cell adhesion and migration necessary for nerve repair. The future direction of peripheral nerve repair is to find a milieu which can support axonal growth through the conduit bridging massive nerve loss. This has led us to the development of bioengineered nerve grafts.

In the present study we propose an alternative to the “gold standard” autologous nerve by using GRG within nerve conduits, which significantly improve peripheral nerve recovery in cases with massive nerve defect.

*Guiding Regeneration Gel (GRG) *was developed to simulate the extracellular milieu and support growth and activity of axons and cells* in vitro* and* in vivo* upon implantation, as well as destined to serve as a regenerative and repair source for nerve tissue reconstruction [12]. The novel GRG has been composed of a highly hydrated, viscous, semisolid gel of high molecular weight (3 × 10^6^ Daltons) hyaluronic acid (HA) and a linear molecule exhibiting antioxidant, anti-inflammatory, healing, repair, and regeneration features. Laminin was added as a synthetic 16 amino acids peptide containing the known cellular active pentapeptides (IKVAV and YIGSR) found in basement membranes which function as adhesive molecules important for mediating and interacting with cytoskeletons elements, integrins, cadherins, cell adhesive molecules (CAMs), and extracellular matrix (ECM) constituents for supporting and guiding cell-neuronal migration, attachment, proliferation, differentiation, survival, regeneration, and growth [13–17]. Similar gels but not the same, especially not with unique features, do exist [18–22]. The third component of the GRG is superoxide dismutase (SOD) for preventing oxidative stress. The combination of HA and SOD is synergistically antioxidant, nonimmunogenic, and anti-inflammatory [23–25].

In* in vivo* experiments performed in peripheral nerve injury model in rats, we compared repair of major nerve loss with GRG in nerve conduit, with empty nerve conduit, with HA in nerve conduit, and with gold standard autologous nerve graft. We found that GRG gel enabled axonal regeneration of 15 mm long nerve gap that was not possible when bridging with an empty tube. GRG gel was shown to enable nerve regeneration at least as good as with autologous nerve graft “gold standard” treatment and enabled significantly enhanced axonal regeneration as compared with HA.

The efficacy of GRG shown with histological methods prompts us to continue our investigation, assessing functional improvement in rats and further establishing GRG capabilities in rabbit experimental model.

## 5. Conclusion 

Utilization of an innovative composite implant to bridge a gap shows promise, suggesting the feasibility of this approach for reconstruction of complete peripheral nerve injury with massive loss defect. GRG gel may serve as a vital component of the bridging station. The proposed technology is expected to provide an alternative to autologous nerve graft.

## Figures and Tables

**Table 1:** 

Number of rats	Treatment of the segmental 15 mm loss∗	Experimental length in time
8	Control operated and further implanted with autogenous peripheral nerve graft	90 days
8	Bridged with standard tube only	90 days
8	Bridged with standard tube and filled with gel vehicle including HA only	90 days
8	Bridged with standard tube filled with GRG	90 days

*In rats model, regeneration within empty tube is possible when gap is up to 7 mm long.
